# SFRP1 reduction results in an increased sensitivity to TGF-β signaling

**DOI:** 10.1186/1471-2407-11-59

**Published:** 2011-02-08

**Authors:** Kelly J Gauger, Kerry L Chenausky, Molly E Murray, Sallie S Schneider

**Affiliations:** 1 Pioneer Valley Life Sciences Institute, Baystate Medical Center, Springfield, MA 01199, USA; 2 Department of Biochemistry and Molecular Biology, University of Massachusetts, Amherst, MA 01003, USA; 3 Department of Biology, University of Massachusetts, Amherst, MA 01003, USA; 4 Department of Veterinary and Animal Sciences, University of Massachusetts, Amherst, MA 01003, USA

## Abstract

**Background:**

Transforming growth factor (TGF)-β plays a dual role during mammary gland development and tumorigenesis and has been shown to stimulate epithelial-mesenchymal transition (EMT) as well as cellular migration. The Wnt/β-catenin pathway is also implicated in EMT and inappropriate activation of the Wnt/β-catenin signaling pathway leads to the development of several human cancers, including breast cancer. Secreted frizzled-related protein 1 (SFRP1) antagonizes this pathway and loss of SFRP1 expression is frequently observed in breast tumors and breast cancer cell lines. We previously showed that when SFRP1 is knocked down in immortalized non-malignant mammary epithelial cells, the cells (TERT-siSFRP1) acquire characteristics associated with breast tumor initiating cells. The phenotypic and genotypic changes that occur in response to SFRP1 loss are consistent with EMT, including a substantial increase in the expression of ZEB2. Considering that ZEB2 has been shown to interact with mediators of TGF-β signaling, we sought to determine whether TGF-β signaling is altered in TERT-siSFRP1 cells.

**Methods:**

Luciferase reporter assays and real-time PCR analysis were employed to measure TGF-β transcriptional targets. Western blot analysis was used to evaluate TGF-β-mediated ERK1/2 phosphorylation. Migration chamber assays were utilized to quantify cellular migration. TERT-siSFRP1 cells were transfected with Stealth RNAi™ siRNA in order to knock-down the expression of ZEB2.

**Results:**

TERT-siSFRP1 cells exhibit a significant increase in both TGF-β-mediated luciferase activity as well as TGF-β transcriptional targets, including Integrin β_3 _and PAI-1. Phosphorylation of ERK1/2 is increased in TERT-siSFRP1 cells in response to enhanced TGF-β signaling. Furthermore, when the TGF-β pathway is blocked with a TGF-βR antagonist (LY364947), cellular migration is significantly hindered. Finally, we found that when ZEB2 is knocked-down, there is a significant reduction in the expression of exogeneous and endogenous TGF-β transcriptional targets and cellular migration is impeded.

**Conclusions:**

We demonstrate that down-regulation of SFRP1 renders mammary epithelial cells more sensitive to TGF-β signaling which can be partially ameliorated by blocking the expression of ZEB2.

## Background

Breast cancer is the most frequently occurring cancer in women and between 12% and 13% of women will develop invasive breast cancer over the course of their lifetime. Cancer results from cellular mutations that enhance proliferation, decrease tumor suppressive signals, and decrease programmed cell death; and from cellular alterations that enhance angiogenesis and metastasis [1]. Notably, metastasis is the most deadly aspect of breast cancer and takes place when invasive epithelial cells in a primary tumor leave their site of origin, digest and break through the extracellular matrix, migrate into blood vessels, and invade secondary sites. Epithelial-mesenchymal transition (EMT) is a process whereby epithelial cells lose polarity as well as cell-cell contacts and undergo a dramatic remodeling of the cytoskeleton resulting in a highly motile mesenchymal morphology. Inappropriate reactivation of EMT is implicated in the conversion of early stage breast tumors into invasive malignancies. Unfortunately, the molecular mechanisms by which EMT mediates the initiation of breast cancer metastasis remain poorly understood.

Transforming growth factor (TGF)-β is a multifunctional cytokine that regulates a variety of physiological processes and also plays a dual role during mammary gland development and tumorigenesis. TGF-β signaling is manifested by way of TGF-β receptor activation and the canonical pathway involves the subsequent phosphorylation of receptor-associated Smad2 and Smad3 proteins which form complexes with Smad4. These activated Smad complexes accumulate in the nucleus where, along with co-activators and cell-specific DNA-binding factors, they regulate gene expression. In early stage breast malignancies, TGF-β functions as a powerful tumor suppressor by blocking cell cycle progression, but TGF-β signaling can become deregulated during mammary tumorigenesis [2,3]. The neoplastic tumor cell environment ultimately transforms TGF-β into an oncogenic cytokine which actively contributes to the acquisition and development of metastatic phenotypes in part through its ability to stimulate EMT and cellular migration [4-7].

Similar to the TGF-β pathway, the Wnt/β-catenin pathway is also implicated in EMT and epithelial plasticity during development and cancer. Wnt ligands activate the Wnt/β-catenin signaling pathway by binding to receptors comprised of Frizzled proteins in conjunction with one of the LDL receptor-related proteins LRP5 or LRP6. Receptor activation results in the ability of a cytoplasmic protein, Dsh, to dissociate and inactivate a multiprotein complex that includes APC, Axin, and GSK3β. As a result, β-catenin is free to accumulate in the nucleus where it forms a complex with the TCF/LEF1 family of HMG box transcription factors and stimulates the expression of specific target genes. Inappropriate activation of the Wnt/β-catenin pathway, which results from mutations in several downstream genes, contributes to the genesis of a wide range of human cancers [8]. However, such mutations are rarely observed in breast cancer despite the finding that β-catenin is stabilized and Wnt signaling is activated in a majority of human breast tumors [9]. In addition, aberrantly activated Wnt signaling leads to inappropriate mammary gland development and mammary tumorigenesis in mice [10].

Secreted frizzled-related protein-1 (SFRP1) is a powerful Wnt signaling antagonist that contains a cysteine-rich domain that is homologous to the Wnt-binding domain of frizzled receptor proteins [11]. Since SFRP1 lacks a transmembrane domain, it is free to remain in the extracellular compartment and block Wnt/β-catenin signaling by binding to Wnt ligands and preventing ligand-receptor interactions [12]. Loss of SFRP1 expression is found in a multitude of cancers including breast cancer [13-15].

When SFRP1 is knocked down in immortalized non-malignant mammary epithelial cells, the cells (TERT-siSFRP1) exhibit a malignant phenotype which resembles the characteristics observed in metastatic breast cancer cells [16]. Interestingly, the phenotypic and genotypic changes that occur in response to SFRP1 loss are consistent with EMT. Among the EMT associated genes analyzed, loss of SFRP1 resulted in a drastic increase in the expression of ZEB2. Originally, ZEB2 was named Smad interacting protein 1 (SIP1) due to it's ability to act in a ligand-dependent fashion with receptor-activated Smads and mediate TGF-β signaling [17]. More recently, studies have revealed that ZEB2 is characterized by N-terminal and C-terminal zinc-finger clusters that can regulate the transcription of EMT markers by binding to 5'-CACCT sequences located in various gene promoters [18]. The work described here was initiated to investigate whether the TGF-β signaling pathway is affected by SFRP1 loss. We demonstrate that down-regulation of SFRP1 renders mammary epithelial cells more sensitive to TGF-β signaling which can be partially ameliorated by blocking the expression of ZEB2.

## Methods

### Cell Culture and Treatments

The 76N TERT cell line (obtained from Dr. Vimla Band) were stably transfected with either pSUPER.retro (TERT-pSUPER) or siSFRP1-PSUPER.retro (TERT-siSFRP1) as previously described [16]. Cells were routinely cultivated at 37°C in 5% CO_2 _and maintained in DMEM/F12 (GIBCO, Grand Island, NY) and the following components from GIBCO: 1% FBS, 1X Antibiotic-Antimycotic (100X), and 20 μg/mL Gentamycin. The following components from Sigma (St Louis, MO) were also used: 50 μM L(+)-Ascorbic acid sodium salt, 1ng/ml Cholera Toxin Vibrio, 12.5 ng/ml Epidermal Growth Factor murine submaxillary, 2 nM β-Estradiol, 0.1 mM Ethanolamine, 1 μg/ml Hydrocortisone-Water Soluble, 1 μg/ml human Insulin solution, 0.1 mM O-Phosphorylethanolamine, 35 μg/ml bovine pituitary extract, 15 nM Sodium selenite, 10 μg/ml human apo-Transferrin, and 10 nM 3,3',5-Triiodo-L-thyronine sodium salt. Cells were plated in 6-well plates (for RNA isolation) and 10 cm dishes (for protein isolation) and allowed to attach overnight. The following day, media was changed to contain vehicle (0.1% BSA/4 mM HCl), 2.5 ng/ml TGF-β1 (Sigma), vehicle (DMSO), 5 μM UO126, and/or 10 μM LY364947 (sigma). For ZEB2 knock down studies, 62.5 nM ZEB2 Stealth RNAi™ siRNA (Invitrogen) was transfected into cells using Lipofectamine™ 2000 (Invitrogen).

### Luciferase Assay

A total of 1 × 10^5 ^cells/well were plated in 24 well plates and were transfected the following day with 0.8 μg CAGA-Luc and 0.08 μg pRL-CMV using lipofectamine ™ 2000 (Invitrogen). After a 6 hour incubation, the media was removed and replaced with treatment media [either vehicle (0.1% BSA/4 mM HCl) or 2.5 ng/ml TGF-β1]. Twenty-four hours after treatment, cells were washed with 1X PBS and lysed using passive lysis buffer (Promega). Luciferase activity was detected using the Dual-Luciferase^® ^reporter assay system (Promega) according to the manufacture's instructions and the light output was measured with a luminometer (TD 20/20 Luminometer, Turner Biosystems, Sunnyvale, CA).

### RNA Isolation and Real-Time PCR

Total RNA was extracted from treated cells using an acid-phenol extraction procedure [19], according to the manufacturer's instructions (Trizol, Invitrogen, Carlsbad, CA). Relative levels of mRNA were determined by quantitative real-time PCR using the Mx3005P™ real time PCR system (Stratagene, La Jolla, CA) and all values were normalized to the amplification of β-Actin. The PCR primer sequences used are as follows: TGF-β2 forward: 5'- CCAAAGGGTACAATGCCAAC -3', TGF-β2 reverse: 5'- CAGATGCTTCTGGATTTATGGTATT-3'; Six1 forward: 5'- ACCGGAGGCAAAGAGACC -3', Six1 reverse: 5'- GGAGAGAGTTGATTCTGCTTGTT -3'; β_3_Integrin forward: 5'- GGGCAGTGTCATGTTGGTAG -3', β_3 _Integrin reverse: 5'- CAGCCCCAAAGAGGGATAAT -3'; PAI-1 forward: 5'- TCCAGCAGCTGAATTCCTG -3', PAI-1 reverse: 5'- GCTGGAGACATCTGCATCCT -3', ZEB2 forward: 5'- AAGCCAGGGACAGATCAGC-3', ZEB2 reverse: 5'- CCACACTCTGTGCATTTGAACT-3', β-Actin forward: 5'-CCAACCGCGAGAAGATGA-3'; β-Actin reverse: 5'-CCAGAGGCGTACAGGGATAG-3'. The assays were performed using the 1-Step Brilliant^® ^SYBRII^® ^Green QRT-PCR Master Mix Kit (Stratagene) containing 200 nM forward primer, 200 nM reverse primer, and 100 ng total RNA. The conditions for cDNA synthesis and target mRNA amplification were performed as follows: 1 cycle of 50°C for 30 min; 1 cycle of 95°C for 10 min; and 35 cycles each of 95°C for 30 s, 55°C for 1 min, and 72°C for 30 s.

### Western Blot Analysis

Treated cells were washed twice with cold PBS and 100 μL of cold lysis buffer [50 mM Tris-HCl, 150 mM NaCl, 100 mM NaF, 10 mM MgCl_2_, 0.5% NP40, protease inhibitor cocktail, and phosphatase inhibitor I and II (Sigma)] was added directly to the plate. The cells were incubated for 30 minutes at 4°C on a shaker and then harvested using a rubber policeman. The lysates were passed 4 times through a 26 gauge syringe, kept on ice for 30 minutes, and then centrifuged for 20 minutes at 12,000 rpms at 4°C. The supernatant was transferred to a new tube and the protein was quantified utilizing the BCA™ Protein Assay Kit (Pierce, Rockford, IL). A total of 50 μg of protein was run on a 10% SDS-Page gel and transferred to a PVDF membrane. The membrane was blocked for 45 minutes with 5% milk in tris-buffered saline containing 0.05% Tween-20 (TBS-T). The primary antibodies used in this study were [Rabbit phospho-ERK1/2 (Thr202/Tyr204) (1:1000), #4377, Cell Signaling Technologies, Danvers, MA; Rabbit ERK1/2 (1:1000), sc-94, Santa Cruz Biotechnology, Santa Cruz, CA; Rabbit β-actin (1:2000), ab8227, Abcam, Cambridge, MA] incubated overnight at 4°C. The secondary antibody [goat anti-rabbit IgG-HRP (Santa Cruz Biotechnology) was applied (1:5000) and incubated for 45 minutes at room temperature. The blot was washed and developed using a Western Blot Luminol Reagent (Santa Cruz Biotechnology).

### Transwell Migration Assays

Treated cells were trypsinized, centrifuged at 1,000 × g for 3 min, and brought to a concentration of 1 × 10^6 ^cells/ml in serum-free media. 5 × 10^5 ^cells/well were seeded in serum free media (containing DMSO or 10 μM LY364947) either in BD BioCoat control chambers (BD Biosciences) above media containing 10% FBS. Following a 22-hour incubation, chambers were removed and cells were fixed for 10 min in 10% formalin, stained for 10 min with 10% Crystal Violet, and rinsed 3X with dH_2_0. Non-migrating were removed from the upper surface of the membrane by scrubbing the insert with a cotton tipped swab moistened with 1X PBS. The insert was then removed from the chamber with a scalpel, and mounted on a microscope slide in Cytoseal™XYL mounting medium (Richard-Allan Scientific). Images were captured with an Olympic BX41 light microscope using SPOTSOFTWARE (Diagnostic Instruments, Inc, Sterling Heights, MI).

## Results

### SFRP1 loss stimulates the TGFβ signaling pathway

**Figure 1:**
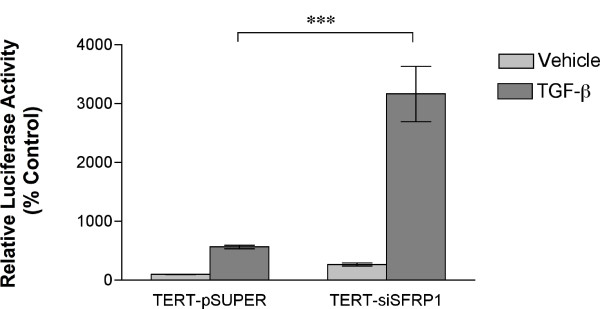
**Loss of SFRP1 enhances TGF-β signaling activity**. TERT-pSUPER and TERT-siSFRP1 cells were transfected with the CAGA-Luc and CMV-Renilla reporter vectors and relative luciferase activity was measured after an overnight incubation in the presence or absence of the 2.5 ng/ml TGF-β1. Bars represent mean ± SEM of relative luciferase activity (firefly luciferase activity/renilla luciferase activity) normalized to the relative luciferase activity in TERT-pSUPER cells treated with vehicle. ***p < 0.001 [significantly different from corresponding TERT-pSUPER cell line using students's *t*-test]

**Figure 2:**
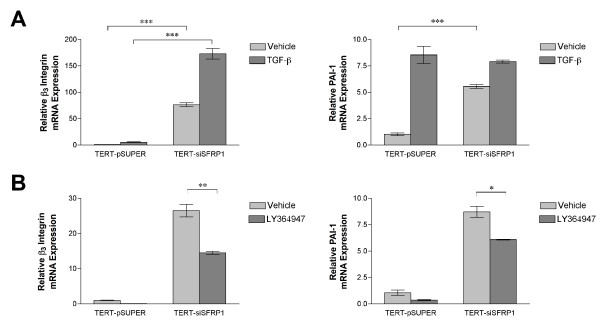
**TGF-β responsive gene expression is augmented in response to SFRP1 loss**. After an overnight treatment with 2.5 ng/ml TGF-β1 (A), or an overnight treatment with 10 μM LY364947 (B), total RNA was isolated from three separate harvests for real-time PCR analysis of β_3 _Integrin and PAI-1. All real-time PCR results are from two separate experiments performed in triplicate and results were normalized to amplification of β-actin mRNA. Bars represent mean ± SEM and are expressed as fold change with respect to TERT-pSUPER cells. *p < 0.05, **p < 0.01, ***p < 0.001 (significantly different from indicated data set using student's *t*-test)

Our initial screen of genes affected by SFRP1 loss in HMECs identified several upregulated genes within the TGF-β pathway, which suggested that there may be an alteration in this signaling pathway in TERT-siSFRP1 cells (Additional File 1). The increased expression of upstream regulators of TGF-β signaling included the TGF-β2 ligand as well as Six1, a homeodomain protein which promotes TGF-β-mediated EMT [20]. Also, mRNA levels of ZEB2 were elevated in TERT-siSFRP1 cells and ZEB2 has been shown to interact with downstream mediators of TGF-β signaling (Smads) and has been suggested to be involved in TGF-β-induced EMT [21]. Finally, Integrin β_3 _and plasminogin activator inhibitor type-1 (PAI-1), which are transcriptional targets of the TGF-β signaling pathway [22,23], were significantly up-regulated in response to loss of SFRP1. Taken together, we directly tested the hypothesis that TGF-β signaling is augmented in TERT-siSFRP1 cells. First, TERT-pSUPER and TERT-siSFRP1 cells were transfected with a TGF-β reporter construct, CAGA-Luc, and grown overnight in the presence or absence of TGF-β1. Twenty-four hours after transfection, luciferase activity was measured and we found that TERT-siSFRP1 cells exhibited a significant increase in relative luciferase activity when grown in the presence of the TGF-β1 ligand (Figure 1). Next, we sought to determine whether endogenous TGF-β responsive gene expression is altered in response to SFRP1 loss. Since both Integrin β_3 _and PAI-1 are transcriptionally upregulated in response to TGF-β1 treatment, we decided to measure the mRNA expression of these genes in TERT-siSFRP1 cells and the effect of TGF-β1 treatment on these genes. The expression of both Integrin β_3 _and PAI-1 was significantly up-regulated in response to SFRP1 loss and treatment with TGF-β1 further increases the mRNA expression levels of Integrin β_3 _in TERT-siSFRP1 cells (Figure 2A, *upper panels*). To verify that the expression of these TGF-β targets was due to aberrant TGF-β signaling in response to SFRP1 down-regulation, cells were treated with a TGF-β receptor (TGF-βR1) specific inhibitor (LY364957) and gene expression was again analyzed by way of real-time PCR. Our results clearly demonstrate that the expression of both Integrin β_3 _and PAI-1 is significantly inhibited in response to LY364947 treatment in TERT-siSFRP1 cells (Figure 2B, *lower panels*).

### Loss of SFRP1 increases the phosphorylation of ERK1/2 through TGF-β signaling

**Figure 3:**
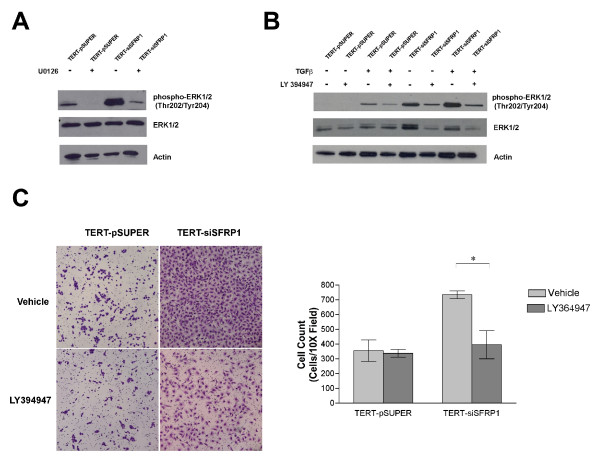
**Loss of SFRP1 increases TGF-β mediated ERK1/2 acivation and migration**. TERT-pSUPER and TERT-siSFRP1 cells were serum starved overnight and treated with 5 μM U0126 (A) or 2.5 ng/ml TGF-β1 and/or 10 μM LY364947 (B). Cell lysates were analyzed for phospho-ERK1/2, ERK1/2, and Actin protein expression by western blot (A-B). TERT-pSUPER and TERT-siSFRP1 cells were plated in BD BioCoat™ control inserts and the cells capable of migrating through the 8 μm pore towards a chemoattractant were stained with 10% crystal violet and counted. Images were captured at 10X magnification (C). Experiments were repeated the number of cells within a representative 10X field from each experiment were counted and bars represent mean ± SEM cell number (D). *p < 0.05, (significantly different from vehicle treated cells using a student's *t*-test).

TGF-β signaling has been shown to increase the phosphorylation of ERK1/2 so we first sought to establish whether of ERK1/2 is phosphorylated in response to loss of SFRP1. Western blot analysis revealed that TERT-siSFRP1 cells express higher levels of phosphorylated ERK1/2 and treatment with an ERK1/2 specific inhibitor (U0126) confirmed that ERK1/2 activation was through a Mek-dependent phosphorylation (Figure 3A, *upper left panel*). We next wanted to determine whether treatment with TGF-β1 could induce the phosphorylation of ERK1/2 in immortalized HMECs and whether the phosphorylation of ERK1/2 we observe in the TERT-siSFRP1 cells could be blocked by antagonizing the TGF-βR with LY364947. Our results illustrate that in our control cell line (TERT-pSUPER), ERK1/2 phosphorylation was observed in response to TGF-β1 treatment and blocking the TGF-βR abrogated this activation. As expected, ERK1/2 was constitutively phosphorylated in TERT-siSFRP1 cells even in the absence of TGF-β1 treatment (Figure 3B, *upper right panel*). Interestingly, treatment with LY364947 reduced the levels of ERK1/2 phosphorylation, independent of TGF-β1 stimulation, which supports the notion that these cells are not only more sensitive to TGF-β, but that TGF-β signaling pathway is also aberrantly activated in TERT-siSFRP1 cells.

TGF-β has been demonstrated to play an important role in activating cellular migration in part by way of ERK1/2 signaling in breast cancer cells [24]. Considering that ERK1/2 is phosphorylated and TGF-β signaling is misregulated in TERT-siFRP1 cells, we tested the hypothesis that the increase in cellular migration observed in response to SFRP1 loss may be partially due to these two aforementioned observations. First, a simple scratch wound assay revealed that TERT-siSFRP1 cells treated with LY364947 were much less motile then cells treated with vehicle (data not shown). These results were confirmed using migration chamber assays. Control cells (TERT-pSUPER) and TERT-siSFRP1 cells were plated in BD BioCoat™ control chambers and the cells capable of migrating through the 8 μm pore towards a chemoattractant were stained with 10% crystal violet and quantified. We clearly show that by blocking the TGF-β pathway with LY364947, we could significantly hinder the migratory action of TERT-siSFRP1 cells (Figure 3C, *lower panel*)

### TGF-β signaling is mediated by ZEB2 in TERT-siSFRP1 cells

**Figure 4:**
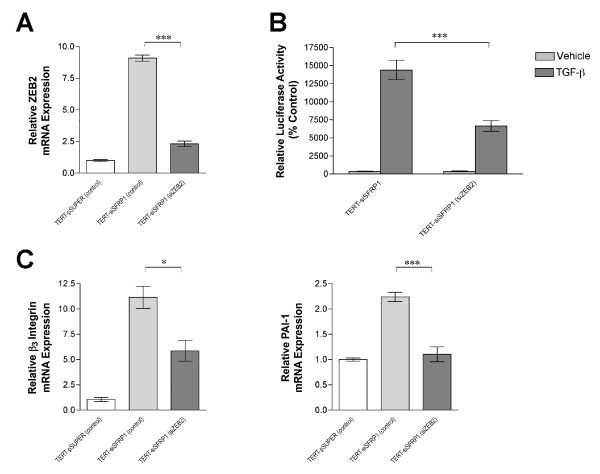
**Knockdown of ZEB2 partially blocks TGF-β signaling in TERT-siSFRP1 cells**. Stealth RNAi™ siRNA negative controls (62.5 nM) were transfected into TERT-pSUPER and TERT-siSFRP1 cells [TERT-pSUPER (control) and TERT-siSFRP1 (control)] and ZEB2 Stealth RNAi™ siRNA (62.5 nM) was transfected into TERT-siSFRP1 cells [TERT-siSFRP1 (siZEB2)]. Forty-eight hours after transfection, total RNA was isolated from each cell line for real-time PCR analysis. The levels of ZEB2 mRNA (A) β_3 _Integrin mRNA and PAI-1 mRNA (C) were normalized to amplification of β-actin mRNA, which was performed in parallel wells for each cell line. Bars represent mean ± SEM and are expressed as relative expression of TERT-pSUPER (control) cells. *p < 0.05, ***p < 0.001 (significantly different from TERT-pSUPER (control) cell line using Bonferroni's *t*-test following 1-way ANOVA). (B) TERT-siSFRP1 cells were transfected with either a Stealth RNAi™ siRNA negative control (62.5 nM) or ZEB2 Stealth RNAi™ siRNA (62.5 nM) and CAGA-Luc plus CMV-Renilla reporter vectors. The relative luciferase activity was measured after an overnight incubation in the presence or absence of the 2.5 ng/ml TGF-β1. Bars represent mean ± SEM of relative luciferase activity (firefly luciferase activity/renilla luciferase activity) normalized to the relative luciferase activity in TERT-siSFRP1 (control) cells treated with vehicle. ***p < 0.001 [significantly different from corresponding TERT-siSFRP1 (control) cell line using students's *t*-test]

**Figure 5:**
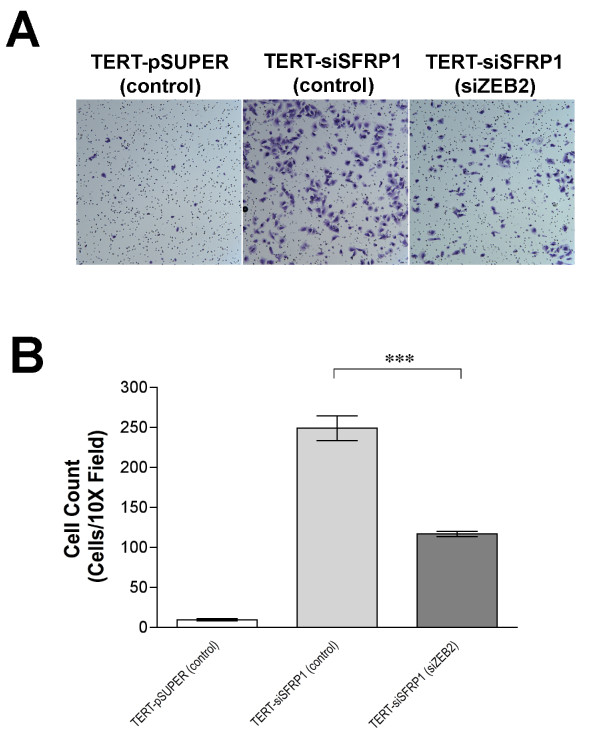
**Knockdown of ZEB2 diminishes the migratory phenotype of TERT-siSFRP1 cells**. Stealth RNAi™ siRNA negative controls (62.5 nM) were transfected into TERT-pSUPER and TERT-siSFRP1 cells and ZEB2 Stealth RNAi™ siRNA (62.5 nM) was transfected into TERT-siSFRP1 cells. After 24 hours, cells were lifted from culture dishes and plated in BD BioCoat™ control inserts and the cells capable of migrating through the 8 μm pore towards a chemoattractant were stained with 10% crystal violet and counted. Images were captured at 10X magnification (A). Experiments were repeated the number of cells within a representative 10X field from each experiment were counted and bars represent mean ± SEM cell number (B). *p < 0.05 (significantly different from TERT-pSUPER (control) cell line using Bonferroni's *t*-test following 1-way ANOVA).

ZEB2 was originally characterized as a Smad interacting protein (SIP) and regulator of TGF-β signaling [17]. We therefore hypothesized that the significant up-regulation of ZEB2 that occurs in response to SFRP1 loss may partially explain the enhanced constitutive activation of TGF-β signaling in TERT-siSFRP1 cells. To test this hypothesis, we knocked-down ZEB2 using siRNAs and real-time PCR analysis verified that there was a significant decrease in the mRNA expression of ZEB2 (Figure 4A, *upper left panel*). Next, TERT-siSFRP1 cells were transfected with control or ZEB2 specific siRNAs, as well as the CAGA-Luc reporter vector, and grown overnight in the presence or absence of TGF-β1. Twenty-four hours after transfection, the luciferase activity was measured. We found that when ZEB2 levels were repressed in TERT-siSFRP1 cells, relative CAGA-luciferase activity was significantly hampered in response to TGF-β1 treatment (Figure 4B, *upper right panel*). In addition, real-time PCR analysis confirmed that the mRNA expression of TGF-β responsive genes, including Integrin β_3 _and PAI-1, was significantly down-regulated when ZEB2 is knocked down in TERT-siSFRP1 cells (Figure 4C, *lower panel*). Finally, we wished to determine whether similar to TGF-βR inhibition, ZEB2 blockade could inhibit cellular migration. Following transfection with control or ZEB2 specific siRNAs, a migration assay using BD BioCoat™ control chambers was carried out and revealed that ZEB2 knockdown reduced the number of TERT-siSFRP1 cells that were capable of migrating through the 8 μm pore towards the chemoattractant (Figure 5A, *upper panel*). The number of cells was quantified and we confirmed that in response to ZEB2 down-regulation, cellular migration was significantly reduced (Figure 5B, *lower panel*).

## Discussion

The present study indicates that loss of SFRP1 expression allows an immortalized non-malignant cell line (76N TERT) to acquire sensitivity to TGF-β signaling due to inappropriate activation of ZEB2. These cells with SFRP1 knocked down (TERT-siSFRP1) display a significant increase in both exogeneous and endogenous TGF-β transcriptional targets, which is further enhanced by the presence of TGFβ. Moreover, ERK1/2 phosphorylation is increased in TERT-siSFRP1 cells by way of enhanced TGF-β signaling. The TGF-β-mediated activation partially explains the migratory phenotype of TERT-siSFRP1 cells considering that when the TGF-β pathway is blocked with LY364947, cellular migration is significantly hindered. Based on our ZEB2 knockdown experiments, the significant up-regulation of ZEB2 that occurs in response to SFRP1 loss partially explains the mechanism by which TGF-β signaling is mis-regulated in TERT-siSFRP1 cells. We clearly show that exogeneous and endogenous TGF-β transcriptional targets, which are upregulated in TERT-siSFRP1 cells, are repressed when the levels of ZEB2 mRNA are decreased. Finally, we found that similar to TGF-βR inhibition, ZEB2 blockade inhibits cellular migration.

Previously, we demonstrated that the Wnt/β-catenin signaling pathway is significantly up-regulated in response to SFRP1 loss [16]. Several different developmental processes and patterning events require inputs from both Wnt and TGF-β family members [25,26]. Work by Labbe *et. al *[27] identified a cohort of genes that are dually affected by both Wnt and TGF-β ligands. Interestingly, several of these genes are elevated in tumors derived from the MMTV-Wnt-1 mouse model and when TGF-β signaling is blocked by overexpression of the dominant negative type II TGF-β receptor, the expression these genes is not only lost, but there is an increase in tumor-free survival. These data indicate that crosstalk between the Wnt signaling pathway and TGF-β cooperate to promote tumor development. Mechanistically, this phenomenon may be partially explained by the finding that Wnt mediated β-catenin translocation results in association with the Lef1 transcription factor, which has been shown to interact with Smad4 and up-regulate TGF-β targets [28]. Thus, the Wnt signaling status of TERT-siSFRP1 likely predicts the TGF-β activity we report here.

The data presented in this article clearly illustrate that TERT-siSFRP1 cells are significantly more sensitive to TGF-β signaling. Not only does TGF-β play a dual role in tumor progression, but it also known to play an important role in human embryonic stem cells [29]. Considering that TERT-siSFRP1 cells exhibit cancer initiating-cell-like characteristics and display a CD44^high^/CD24^low ^cell surface marker expression pattern [16,30], our current results are fully consistent with previously published research by Shipitsin *et. al *showing that CD44^high^/CD24^low ^cells are more responsive to TGF-β and can be specifically targeted by TGF-βR inhibition [31]. These data support the notion that the Wnt/β-catenin and TGFβ signaling pathways are feeding into each other and facilitating the development of aggressive tumor characteristics.

Downstream transcriptional targets of TGF-β, including Integrin β_3 _and PAI-1 are also significantly more sensitive to TGF-β when the expression of SFRP1 is knocked down. The finding that Integrin β_3 _is upregulated in TERT-siSFRP1 partially explains the migratory and invasive characteristics exhibited by these cells. When breast cancer cells express Integrin β_3_, their invasiveness is enhanced *in vivo *[32]. Also, it has previously been shown that the induction of Integrin β_3 _expression during mammary tumorigenesis correlates with the development of metastases and that when Integrin β_3 _is antagonized, breast cancer cell invasion abolished [33]. Consistent with what we present in this current report, Galliher *et. al *demonstrate that the expression of Integrin β_3 _alters the response of mammary epithelial cells to TGF-β-mediated EMT [33]. Interestingly, small molecule inhibitors of Integrin β_3 _are currently being developed as anti-cancer therapeutics [34]. Likewise, PAI-1 is also a proposed anti-cancer target [35]. Several components of the plasminogen activation system, including PA1-1, are implicated in tissue remodeling associated with both physiological and pathologic processes such as wound healing, mammary gland involution, angiogenesis, and cancer invasion [36]. Specifically, PAI-1 promotes tumor growth, angiogenesis, and metastasis [37-40]. Our findings show that by reducing ZEB2 levels, we can subsequently reduce the expression of both Integrin β_3 _and PAI-1. Taken together, these data suggest that ZEB2 is a central transcriptional regulator, which controls gene expression necessary for the progression of metastatic cancer. Elloul *et. al. *[41] analyzed the expression of ZEB2 as well as other transcriptional repressors of E-cadherin in ovarian and breast carcinoma effusions and revealed that elevated mRNA levels of ZEB2 are a predictor of poor survival [41]. Thus, ZEB2 may be an important anti-cancer therapeutic target.

In order to establish whether TGF-β signaling elicits a direct physiological consequence when SFRP1 is downregulated, we measured activated ERK1/2 levels and the migratory action of in TERT-siSFRP1 cells in response to TGF-βR inhibition. As hypothesized, when cells were treated with LY364947, ERK1/2 phosphorylation and cellular migration were drastically reduced. Our results are consistent with previous research which has shown that TGF-β signaling mediates the cellular migration of breast cancer cells by several pathways including ERK1/2 activation [42]. Interestingly, the mechanism by which TGF-β phosphorylates ERK1/2 is independent of Smad4, as evidenced by TGF-β induced phosphorylation of ERK1/2 in the Smad4 deficient MDA-MB-468 cell line [24]. Our data would agree with this observation. Knock down of ZEB2 did not affect the levels of phosphorylated ERK1/2 (data not shown), however inhibition of the TGF-βR did affect the ERK1/2 phosphorylation observed in TERT-siSFRP1 suggesting that loss of ZEB2 affects the canonical TGFβ-Smad pathway, but not other TGF-β initiated pathways. Alternatively, the phosphorylation of ERK1/2 in TERT-siSFRP1 cells may require input from multiple signaling pathways. Since ZEB2 is likely augmenting TGF-β signaling by way of its Smad interacting domain, the mechanisms by which the TGF-β pathway affects cellular migration in TERT-siSFRP1 cells seems to involve both Smad-independent (ERK1/2) and Smad-dependent (ZEB2) actions.

It has been suggested that overexpression of ZEB2 plays a putative role in oncogenic transformation since it was identified in a large-scale screen for cancer related genes [43]. Data illustrating that high levels of ZEB2 are expressed in migratory breast cancer cell lines (MDA-MB-231 and MDA-MB-435S1) but not in breast cancer cells with a more epithelial/less migratory phenotype (MCF7/AZ) [44] supports our finding that ZEB2 repression hampers the cellular migration. It is thought that the mechanism by which ZEB2 induces EMT is by its ability to repress the epithelial cell junction protein E-cadherin and induce the mesenchymal marker, vimentin, in breast cancer cell lines [45]. However, the decrease in cellular migration that occurs in response to ZEB2 down-regulation in TERT-siSFRP1 is independent of E-cadherin and vimentin, as the mRNA levels of these genes are only marginally affected by ZEB2 knockdown (data not shown). The down-regulation of E-cadherin in TERT-siSFRP1 cells is likely due to the misregulation of a combination of E-cadherin transcriptional factors including ZEB1, Twist, Snail, and Slug. The expression of vimentin may be mediated by β-catenin in TERT-siSFRP1 cells since β-catenin can transactivate vimentin in breast cancer cells [46] and β-catenin activity is enhanced in TERT-siFRP1 cells [16]. Taken together, this research suggests that a reduction of SFRP1 can sensitize immortalized mammary epithelial cells to enhanced migration through an increased activation and sensitivity of the TGF-β signaling pathway.

## Conclusions

This is the first report to suggest that the loss of a Wnt signaling antagonist, SFRP1, plays a direct role in TGF-β-mediated signaling and TGF-β-mediated-cellular migration. Given our results, the crosstalk that occurs between the Wnt/β-catenin and TGFβ signaling pathways seems to be in part synchronized by SFRP1. Experiments to elucidate whether TERT-siSFRP1 cells are tumorigenic *in vivo *and how TGF-β targets are affected by the paracrine signals elicited are ongoing. In addition, this work described here is the first to demonstrate TGF-β sensitivity can be partially ameliorated by blocking the expression of ZEB2, a key transcription factor that regulates the progression of tumor cell metastasis. Hence, ZEB2 may be an excellent anti-cancer therapeutic target.

## Abbreviations

EMT: epithelial-mesenchymal transition; TGF-β: transforming growth factor-β; LDL: low-density lipoprotein; Dsh: Dishevelled; APC: Adenomatous polyposis coli; GSK3: Glycogen synthase kinase 3; HMG: high mobility group; SFRP1: Secreted frizzled-related protein-1; SIP1: smad interacting protein 1; ZEB2: Zinc finger E-box-binding homeobox 2; PAI-1: plasminogin activator inhibitor type-1; plasminogin activator inhibitor type-1; ERK1/2: extracellular-signal-regulated kinase1/2.

## Competing interests

The authors do not have any financial or personal relationships with other people or organizations that could inappropriately influence the work described in this manuscript.

## Authors' contributions

KG drafted the manuscript and performed all of the described experiments. KC treated cells and harvested RNA and protein utilized for the described experiments. MM imaged the migration assay slides, counted cells, and quantified the results. SS participated in the study design, edited the manuscript, and gave final approval of the version to be published. All authors read and approved the final manuscript.

## Pre-publication history

The pre-publication history for this paper can be accessed here:

http://www.biomedcentral.com/1471-2407/11/59/prepub

## Supplementary Material

Additional File 1 **Genes associated with the TGF-β signaling pathway are up-regulated in response to SFRP1 down-regulation**. Total RNA was isolated from three separate harvests for real-time PCR analysis of genes involved in the TGF-β signaling pathway. All real-time PCR results are from two separate experiments performed in triplicate and results were normalized to amplification of β-actin mRNA. Bars represent mean ± SEM and are expressed a fold change with respect to TERT-pSUPER cells. **p < 0.01, ***p < 0.001 (significantly different from corresponding TERT-pSUPER cell line using students's *t*-test) Click here for file
