# Supplementary figures and images for: SFRP1 reduction results in an increased sensitivity to TGF-β signaling
Source: BMC Cancer. 2011 Feb 8;11:59. doi: 10.1186/1471-2407-11-59 (PMC3041779; doi:10.1186/1471-2407-11-59)

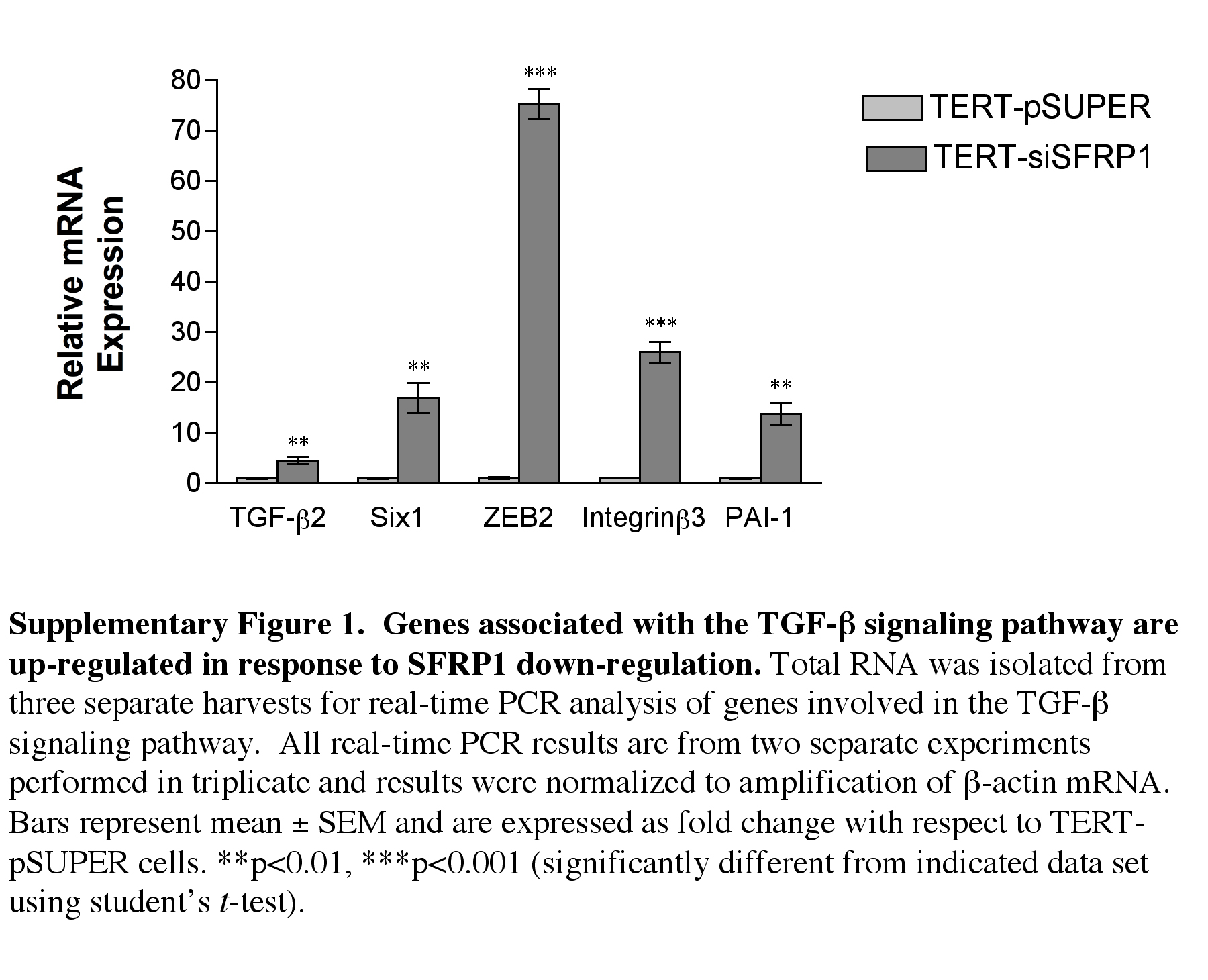

Supplement: Additional File 1 — Genes associated with the TGF-β signaling pathway are up-regulated in response to SFRP1 down-regulation. Total RNA was isolated from three separate harvests for real-time PCR analysis of genes involved in the TGF-β signaling pathway. All real-time PCR results are from two separate experiments performed in triplicate and results were normalized to amplification of β-actin mRNA. Bars represent mean ± SEM and are expressed a fold change with respect to TERT-pSUPER cells. **p < 0.01, ***p < 0.001 (significantly different from corresponding TERT-pSUPER cell line using students's t-test) [file 1471-2407-11-59-S1.JPEG]
